# Current state and advancements of imaging in acute ischemic stroke: a practical review

**DOI:** 10.1007/s10072-025-08685-8

**Published:** 2025-12-22

**Authors:** Ali Al-Salahat, Yasaman Pirahanchi, Thirumalaivasan Dhasakeerthi, Divya Nayar, Sama Almasri, Khushboo Verma, Abhilash Thatikala, Mohammed Alkhaldi, Seyed Amir Ebrahimzadeh, Nastaran Shahsavari, Raja Godasi, Nidhi Kapoor, Rohan Sharma

**Affiliations:** 1 Department of Neurology, Creighton University, Nebraska, USA; 2 Department of Neurology, UC San Diego, San Diego, CA USA; 3 Department of Neurology, University of Arkansas Medical Center, Little Rock, AR USA; 4 Department of Radiology, Creighton University, Nebraska, USA; 5 Department of Neurology, St. Luke’s Boise Medical Center, Idaho, USA

**Keywords:** Neuroimaging, Stroke, Acute ischemic stroke, Imaging modalities, Stroke imaging

## Abstract

**Background:**

Neuroimaging has a pivotal role in the diagnosis, management, and prognostication of acute ischemic stroke (AIS). Developments have improved the ability to identify large vessel occlusions (LVO), delineate salvageable tissue, and guide acute therapeutic interventions. This review aimed to provide an overview of neuroimaging modalities used in AIS evaluation, highlighting their practical applications, recent advancements, and limitations.

**Methods:**

We conducted a narrative review of the literature, focusing on non-contrast CT (NCCT), CT angiography (CTA), CT perfusion (CTP), MRI, MR angiography (MRA), digital subtraction angiography (DSA), and transcranial Doppler ultrasonography (TCD). Additionally, we examined emerging trends, such as virtual NCCT, sonothrombolysis and direct-to-angio suite (DTAS) approach. Key aspects, including diagnostic accuracy, clinical utility, and emerging technologies were evaluated.

**Findings:**

NCCT remains the cornerstone for initial AIS assessment due to its availability and its ability to assist in rapid decision making. CTA and CTP are integral for detecting LVO and assessing penumbra, respectively, with automated systems enhancing speed and accuracy. MRI helps identify early ischemic changes and rule out stroke mimics. MRA and DSA provide detailed vascular assessments, while TCD offers hemodynamic monitoring and a potential therapeutic role. Limitations include variability in perfusion imaging, MRI availability, and operator dependency in TCD. Emerging techniques like dual-energy CT and AI-driven algorithms enhance diagnostic precision.

**Conclusion:**

Neuroimaging is essential for effective AIS management, with each modality carrying variable strengths and limitations. Continued advancements, standardization and a multi-modal approach are needed to optimize outcomes. Clinical-radiological correlation remains indispensable to reach accurate diagnosis and treatment.

## Introduction

Ischemic stroke still carries a marked burden on global public health as a major cause of disability and mortality [1, 2]. Imaging plays an integral part in the diagnosis, management and prognostication of acute ischemic stroke [3]. Indeed, the ultimate goal of stroke imaging is to identify patients who are candidates for reperfusion therapies (i.e. thrombolysis and thrombectomy). Non-contrast CT is most commonly the first imaging modality in acute ischemic stroke (AIS), assisting initial decision-making for administering thrombolysis [4]. CT angiography and perfusion are also part of the initial “acute stroke protocol CT” in most centers, guiding selection for endovascular intervention [3, 4]. MRI additionally helps to confirm the diagnosis in some cases, rule out stroke mimics and direct acute intervention in patients presenting outside the conventional “window” for treatment [5]. Interestingly, machine-learning and artificial intelligence are increasingly being utilized in acute ischemic stroke imaging [6, 7]. This review aims to focus on the most important practical aspects of imaging in AIS, describing the modalities involved and recent advances. First, we will review the long-standing pillar of acute stroke imaging: non-contrast CT (NCCT).

### Non-contrast CT

#### Role in thrombolysis

NCCT has been a cornerstone in the evaluation of AIS since its introduction in the 1980s. Its widespread availability, safety, and rapid execution make it an indispensable tool in the acute management of patients with AIS. NCCT has several important utilities regarding AIS evaluation and management. The main initial objective of NCCT in AIS is to rule out hemorrhage and determine eligibility for thrombolysis [8]. It can rapidly exclude some of the other causes of acute neurological symptoms [9].

#### Early ischemic signs

**Fig. 1:**
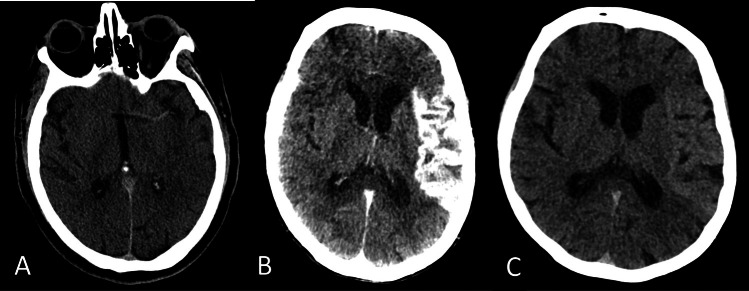
A. Non-contrast head computed tomography (CT) demonstrates a linear hyperdensity at the expected location of the left middle cerebral artery (MCA) consistent with acute thrombus, "hyperdense MCA" sign. **B**. Dual Energy head CT in a patient with left MCA territory stroke, status post endovascular intervention. Mixed image (left) shows gyriform pattern of intense hyper-attenuation involving the left insular and left temporal-frontal-parietal cortices. Virtual unenhanced image (right) shows mild hyper-attenuation and gray-white matter differentiation loss of the left MCA territory which is suggestive of blood brain barrier breakdown, neovascularization and impaired autoregulation. There was no hemorrhagic transformation

NCCT can be used to evaluate for the immediate radiological features of AIS [10–13]. The hyperdense middle cerebral artery sign (HMCAS) is an appearance of increased attenuation of the proximal middle cerebral artery (MCA) that is often associated with thrombosis of the M1 MCA segment (Fig. 1) [10]. This has a sensitivity of 67% and specificity of 82% for detecting large vessel occlusion (LVO) [10, 11]. Similarly, other arteries can show the hyperdense sign on NCCT, such as basilar artery and internal carotid artery, albeit less common than HMCAS [14, 15]. Indeed, the hyperdense basilar artery sign can be crucial to recognize as the clinical presentation of basilar artery occlusion (BAO) can be vague, non-specific and challenging and, hence, delayed [16, 17]. A hypodense artery sign is another rare and less recognized finding on NCCT that is associated with fat or air embolism [18].

Loss of the insular ribbon sign, which is the loss of grey-white differentiation of the lateral margin of the insular cortex secondary to early cytotoxic edema, can be associated with large infarction and poor outcomes [12]. Early ischemic changes in the basal ganglia, identified as obscuration of the Lentiform nucleus, have lower sensitivity at 48% for an associated deep subcortical infarct [12, 13]. Cortical infarcts may be detected via sulcal effacement with a sensitivity of 69% and can also be associated with poor outcomes [12]. Additionally, NCCT can be of important utility in the early hyperacute phase (6 to 24 h) by detecting loss of differentiation between the white and gray matter. This detection is optimized via the “stroke window” by using variable window width and center level settings (center level of 32 Hounsfield Units and width of 8 Hounsfield Units) [19].

#### Alberta stroke program early CT score

**Fig. 2:**
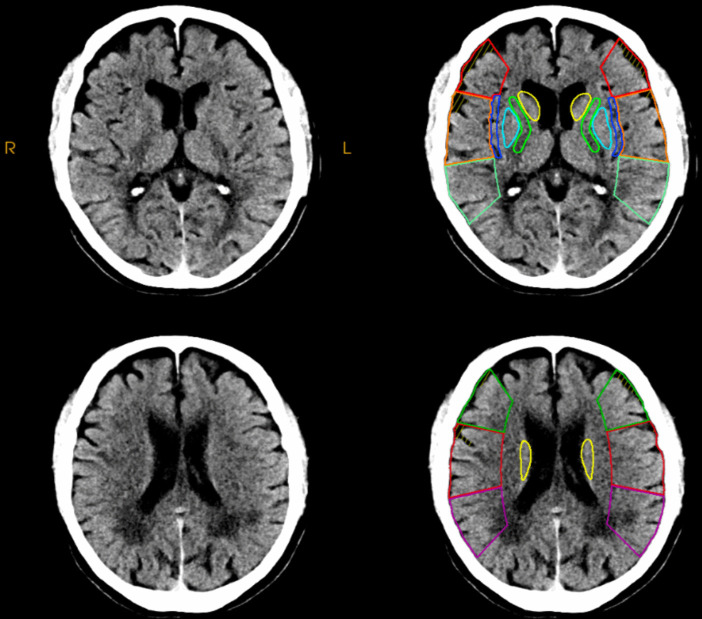
Non-contrast head computed tomography (CT) depicting the Alberta Stroke Program Early CT Score (ASPECTS)

NCCT is essential for calculating the Alberta Stroke Program Early CT Score (ASPECTS) that guides acute stroke intervention (Fig. 2) [20]. ASPECTS is a scoring system based on 10 regions of the brain that are most affected in an ischemic stroke in the MCA territory. Each of these regions is given a score of 1 point if normal and 0 points if damaged by ischemia [20]. A lower score indicates a worse prognosis and a larger area of brain damage. ASPECTS also helps guide clinical decisions for the treatment of stroke patients, such as the consideration for thrombectomy or thrombolysis. A posterior circulation ASPECTS is also being increasingly recognized, but it requires further validation at this point [20]. Additionally, NCCT readily identifies early signs of malignant cerebral edema and mass effects including effacements of sulci and lateral ventricles or midline shift [21].

#### Limitations

NCCT still has several limitations. Large cortical infarcts might not be detected by NCCT up to 3 h from onset, and around 40% of the infarcts can be missed by 24 h [9]. Therefore, accurate estimation of the age of infarct is extremely limited using NCCT alone. However, recent advanced and promising techniques utilizing net water uptake on CT showed “MRI-level” accuracy in identifying infarcts within 4.5 h [22]. Given the anatomical complexity and density of the bony structures in the brainstem and cerebellum with resultant artefacts, NCCT has low sensitivity reaching 41.8% in the posterior fossa [23]. Moreover, the calculation of ASPECT score using NCCT can have inter-rater variability limiting its reliability [24]. Hence, automated calculation of ASPECT score is increasingly utilized to improve reliability and consistency [25]. Another interesting pitfall relating to the use of NCCT in ischemic stroke is the CT fogging phenomenon. The initial hypodense infarct apparent on NCCT can become iso-dense or even invisible in the 2–3-week period after an ischemic stroke [26]. This fogging phenomenon, if not recognized, can lead to underestimation of the size of the infarct. It is believed to occur due to the clearing of the necrosis and the development of angiogenesis within the infarct [27].

### Dual-energy CT

#### Differentiating contrast-staining from hemorrhage

Dual-energy CT (DECT) can be a useful tool to differentiate tissues with similar X-ray attenuation, such as contrast and hemorrhage (Fig. 1) [28, 29]. This advantage is crucial when patients receive iodinated contrast prior to the CT, such as after endovascular stroke intervention [28, 29]. By using two distinct X-ray energy levels to acquire data, DECT can differentiate between contrast-staining due to blood–brain barrier breakdown and hemorrhage [30]. DECT also improves detectability of early ischemic changes in AIS [28–30].

#### Virtual non-contrast CT

Virtual non-contrast CT (VNCCT), which is reconstructed from DECT angiography, is increasingly being recognized as a suitable alternative to conventional NCCT for the evaluation of AIS [31]. It allows the acquisition of both vascular and parenchymal evaluations using a single image, streamlining the AIS imaging workflow [32]. Studies showed that VNCCT can be non-inferior to conventional NCCT, but guidelines still recommend the latter as the initial imaging technique [31, 32].

### CT Angiography

**Fig. 3:**
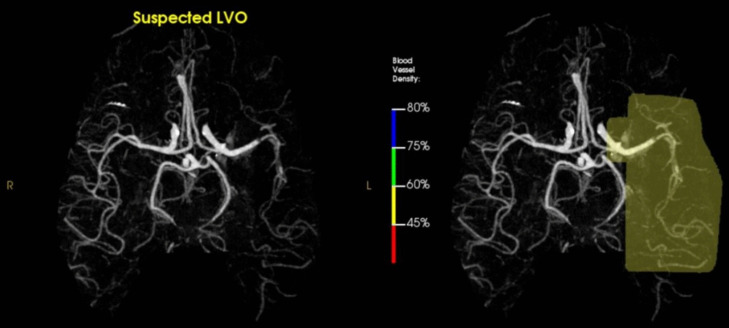
Reconstructed 3D images of an intracranial computed tomography (CT) angiography demonstrating RAPID-software detecting large vessel occlusion

CT angiography (CTA) of the head and neck has become part of the standard “stroke protocol” imaging given its rapid acquisition, availability and accuracy in identifying LVO to guide acute stroke intervention (Fig. 3) [33]. After the DAWN and DEFUSE 3 trials extended the window for endovascular thrombectomy (EVT), “CTA-for-All” protocol became standard in many institutions [34]. The sensitivity and specificity of CTA are around 91% and 93% for anterior LVO, but decrease to 73% and 92% for all LVOs, respectively [35]. However, for medium vessel occlusion (MeVO), the sensitivity and specificity of CTA is around 65% and 93% [35]. CTA provides detailed definition of the vasculature of extracranial and intracranial arterial circulation. This helps in diagnosing a variety of vascular lesions that can be associated with AIS, such as dissection, stenoses, and aneurysms. Additionally, it can help identify anatomical variants in the extracranial or intracranial circulation. Even though Digital Subtraction Angiography (DSA) is considered the gold standard in evaluating the vasculature in stroke, studies showed that CTA had a sensitivity of 97.1% and specificity of 99.5% for detecting 50% stenosis in large intracranial arteries in comparison to DSA [36]. Indeed, other studies showed that CTA can outperform MRI angiography (MRA) for intracranial stenoses and occlusions with higher sensitivity and positive predictive value (98% vs 70%, 93% vs 65%, respectively) [36, 37]. Moreover, studies showed that CTA can be a highly sensitive test for extracranial carotid arterial stenosis as compared to DSA and serves as an excellent screening tool [38].

Several recent advancements in CTA techniques enhanced its use in AIS. Automated detection systems have been employed for rapid and reliable detection of LVO (98% specific and 96% sensitive), with some high-performance systems averaging 3 min and 18 s for processing the images [39]. Deep learning-based algorithms have been utilized to provide a focused view CTA which can hasten the LVO detection [40]. Multiphase CTA (mCTA) also improved the detection of LVO and the assessment of collateral circulation by capturing multiple phases of enhancement [41]. To enhance the visualization of the vasculature using mCTA, time-variant color maps have been incorporated to ease the evaluation of the collateral circulation and detection of distal occlusions [42]. Spectral CT (SCT) is another technique that can distinguish core of the infarct from the penumbra, potentially being an alternative to CT perfusion (CTP) while reducing the radiation and contrast involved [43].

CTA-ASPECT is an extension of the ASPECTS used to evaluate ischemic stroke on CTA. Calculating ASPECTS on CTA source images (CTA-ASPECTS) has been shown to be a superior predictor of final infarct size and clinical outcome compared to NCCT-ASPECTS [44]. The advantage of using CTA source images or CTA maximum intensity projection images in calculating ASPECTS comes from the fact that it considers and directly shows the degree of collateral circulation and better delineates the infarct territory [44].

CTA has some limitations that can have negative consequences on the evaluation and management of LVO in AIS. Arterial phase CTA in patients with major intracranial occlusion can lead to false non-attenuation of the proximal cervical arteries, especially the cervical carotid artery, a phenomenon known as pseudo-occlusion [45]. It is likely due to the swift acquisition of CTA which surpassed the comparatively sluggish flow of contrast in the arteries with distal occlusion. A study showed that there was lack of interobserver agreement among radiologists when it pertains to carotid artery pseudo-occlusion [46]. This may ultimately affect decision making in acute stroke intervention. mCTA can be helpful in distinguishing pseudo-occlusion from a true occlusion as it captures multiple enhancement phases [47]. CTA can also be limited in evaluating distal intracranial occlusions or MeVO [35]. Radiation exposure can pose a problem especially when thinner slices are required for detailed images. Finally, recent evidence suggests that contrast-induced nephropathy is less of a risk than previously believed [48, 49].

### MRI

#### Strengths of MRI in AIS

**Fig. 4:**
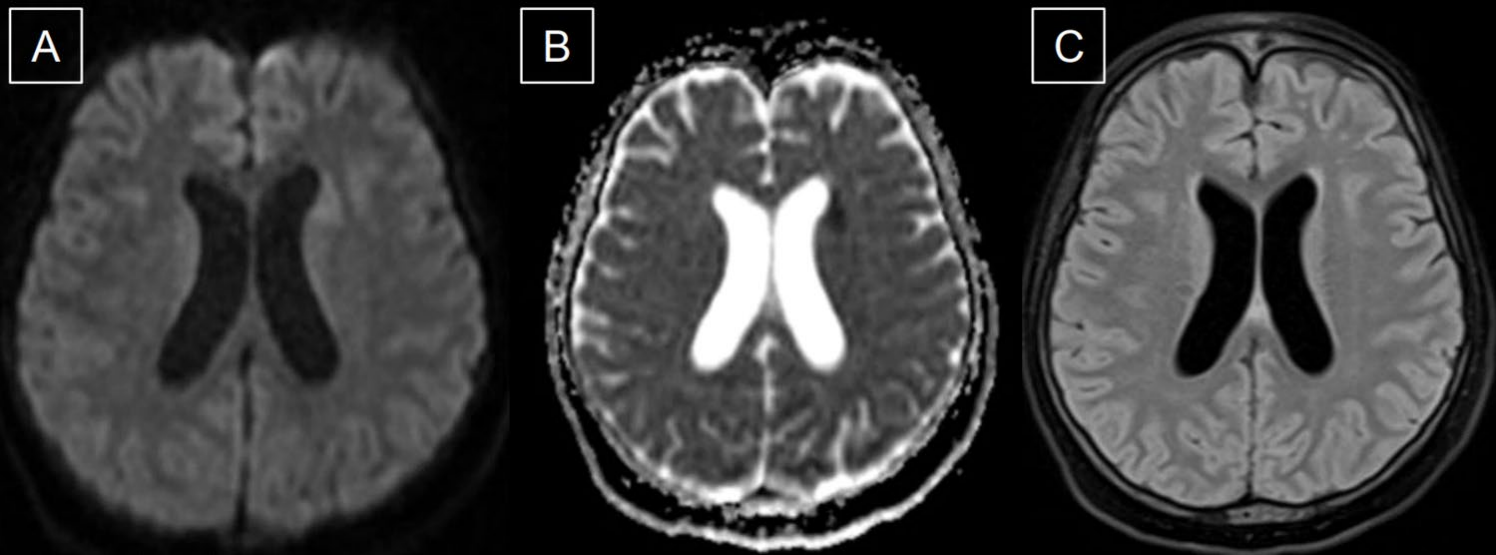
Diffusion-weighted magnetic resonance (MR) image of the brain (**A**), demonstrates a small area of hyperintensity in the anterior left periventricular white matter, which corresponds with hypointensity on the apparent diffusions coefficient (ADC) map (**B**). Of note, there is no signal change on the fluid-attenuated inversion recovery (FLAIR) image (**C**) in this acute infarct

Brain MRI with its multiple sequences, which exploit different tissue properties, shows superiority in ruling out stroke mimics, detecting AIS and determining onset and severity [50]. A 6-min MRI stroke protocol has been suggested to be a feasible tool in the evaluation of AIS, and it includes DWI, ADC, fluid-attenuated inversion recovery (FLAIR), gradient recall echo (GRE), MRI angiography (MRA), and perfusion MRI [51]. Additionally, MRI can be used to extend the reperfusion window beyond the conventional time-based window to tissue-based window, utilizing DWI and perfusion-weighted imaging (PWI) to delineate viable tissue (Fig. 4) [52, 53]. This is particularly helpful in “wake-up” AIS and in cases where exact time of onset is unclear.

#### Temporal MRI changes in AIS

In the early hyperacute phase of AIS (i.e. within minutes), diffusion-weighted imaging (DWI) begins to show an increased signal and reduced apparent diffusion co-efficient (ADC) signal as cytotoxic edema restricts the diffusion of water [54]. Other early hyperacute AIS signs on MRI are susceptibility vessel sign on susceptibility-weight imaging (SWI) and loss of flow void with hyperintense signal on T2/FLAIR due to stagnant or slow flow [55]. Indeed, a prominent vessel sign on SWI can be an indicator of poor collateral circulation and poor functional outcomes [56]. Proceeding into the late hyperacute phase (i.e. after 6 h), T2 hyperintense signal starts to appear which continues over the first few days, while T1 hypo-intensity usually manifests after 16 h of onset [54]. T1 contrast enhancement in AIS can be divided into two types. Intravascular enhancement is most seen within 24–48 h after AIS but can appear as early as one hour after a stroke and persist for around a week [56]. This pattern of enhancement is due to slow flow within the vessel or stasis [56]. Parenchymal enhancement due to the breakdown of blood–brain barrier can remain for several weeks after AIS [56, 57].

#### MRI-based aspects

Several studies have examined the reliability and accuracy of MRI-ASPECTS in comparison to CT-ASPECTS. McTaggart et al. showed that inter-rater agreement was higher with MRI-ASPECT than CT-ASPECTS [58]. MRI-ASPECTS or DWI-ASPECTS also showed to be more predictive of functional outcomes and infarct volume than CT-ASPECTS [20].

#### Limitations of MRI in AIS

Limitations relating to the use of MRI in AIS are mainly owing to availability and time of acquisition. Recent evidence shows that MRI use in AIS can be associated with lower thrombolysis rates and delay in door-to-needle times, but not with thrombectomy related outcomes [59]. Patients with metallic devices or objects can also represent some of the limitations relating to the use of MRI in AIS. Data suggest that improving access to MRI on a 24/7 basis in suspected AIS can help reduce hospital admissions and lower length of hospital stays [60, 61].

#### MRI-negative stroke

An interesting phenomenon that might pose a challenge to neurologists is MRI-negative stroke or DWI-negative stroke. This entity has been shown to carry better functional outcomes, and lower odds of disability or mortality, but it requires good clinical judgment to diagnose [62]. A systematic review reported MRI-negative stroke in around 16% of stroke patients, while another study estimated its prevalence to be around 6.8% [62, 63]. DWI-negative stroke is more common when the posterior circulation is involved and with minor stroke (i.e. NIHSS 5 or less) [63].

#### Post-intervention findings

Sulcal focal subarachnoid hemorrhage is a sign that can been on MRI in the setting of AIS, especially after thrombectomy due to reperfusion injury and rupture of the small cortical vessel [64, 65]. Sulcal focal subarachnoid hemorrhage can also be evident on NCCT and should not raise concern as it is a benign finding and might in fact indicate favorable outcomes [65].

### CT perfusion and MRI perfusion

#### Principles and parameters

Both CTP and MRI perfusion (MRP) are functional imaging modalities that are used to evaluate salvageable brain tissue, differentiating penumbra from the infarct core. CTP is increasingly being adopted as part of the standard “stroke protocol” CT, as it is readily available and rapidly acquired [66]. Moreover, studies showed that incorporating CTP into the stroke protocol increased the rates of LVO detection and, hence, thrombectomy rates [67]. Obtaining CTP involves continuously imaging a single location during the contrast bolus, which consists of 40–50 mL at 3–5 mL/s, during its transit throughout the cerebral vessels [68]. The data is then processed to generate color-coded maps via an automated software that mainly yields cerebral blood flow (CBF), cerebral blood volume (CBV), and mean transit time (MTT) [66, 68]. While CBV is basically the volume of blood in a given amount of brain tissue, CBF is volume of blood passing through a given area of brain tissue per unit time [68]; MTT is the average time for blood to pass through a given amount of brain tissue [68].

#### Advantages of Tmax

**Fig. 5:**
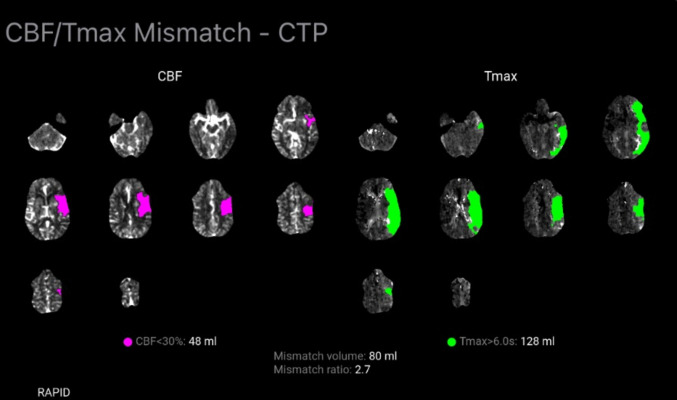
Computed tomography (CT) perfusion of the brain demonstrates significant mismatch volume secondary to a left middle cerebral artery (MCA) infarct. On the left images, brain areas with less than 30% cerebral blood flow are shown in purple; on the right panel, areas with Tmax more than 6.0 s are shown in green. Mismatch volume of 80 ml demonstrates considerable amount of salvageable brain tissue for endovascular intervention

Time to maximum (Tmax) is the time delay (in seconds) between the arrival of the contrast bolus in a major artery and its peak concentration in the brain tissue [69]. It is a sensitive measure of perfusion delay [69]. It indicates ischemic penumbra as it measures the time from arterial input function to the point of maximum concentration in the tissue residue function (Fig. 5) [70]. Tmax can be easily interpreted even by less experienced readers with high diagnostic accuracy, making it suitable for AIS evaluation and management [70]. Other practical advantages of Tmax include triaging patients with distal MeVO for endovascular intervention [71]. It can also be used to predict risk for hemorrhagic transformation after thrombolysis indicated by a Tmax higher than 14 s [72]. A cut-off of 6 s has been shown to be highly sensitive and specific for identifying penumbra using Tmax obtained by CTP or MRP [70]. Overall, the penumbra shows increased Tmax/MTT, decreased CBF and normal/increased CBV, while the infarct core shows increased Tmax/MTT and markedly reduced CBF and CBV (Fig. 5).

#### MR perfusion versus CT perfusion

The conventional method used to obtain MRP is dynamic susceptibility contrast (DSC) which yields the same hemodynamic parameters as CTP, including CBV, CBF and MTT [73]. Arterial spin-labelling is another MRP technique that is advantageous in not requiring contrast to obtain perfusion parameters [73]. Data obtained from MRP can be similar to CTP, with the former having a few advantages and disadvantages. MRP can obtain images with higher resolution especially in the brainstem and cerebellum and it does not involve ionizing radiation. However, it is much more difficult to access and obtain compared to CTP.

#### Limitations and pitfalls

There are several limitations pertaining to perfusion imaging in AIS. First, there exists consequential variability in the use of CTP in AIS. A recent study analyzed variability in CTP among stroke centers, finding significant differences in ischemia estimation [74]. The primary source of this variability was found to be the perfusion vendor software algorithm rather than acquisition method [74]. Future work needs to focus on unification and standardization of perfusion software algorithms. Overestimation of the infarct core, otherwise known as ghost infarct core, has been frequently reported in AIS [75]. This can impact decision making and lead to inappropriate exclusion of patients from reperfusion therapies. Relatedly, Tmax and MTT can sometimes overestimate the penumbra, because they are unable to differentiate between areas of oligemia and ischemia, leading to unnecessary intervention in AIS [73]. Lastly, perfusion imaging can be difficult to interpret in patients with low cardiac output, which leads to global hypoperfusion, or in patients suffering from seizures, with post-ictal state mimicking stroke [76].

### MRI angiography

#### MRA versus CTA

Both MRA and CTA can provide essential data on the intracranial and extracranial vasculature in the evaluation and management of AIS. Even though CTA is often preferred in the initial evaluation of AIS, MRA can still be used in the “acute stroke MRI protocol” depending on availability. The main advantage of MRA in general is that it can be incorporated into other MRI-based sequences that can yield more data than CT-based modalities in AIS, especially with smaller infarct volumes.

#### Comparing MRA techniques

**Fig. 6:**
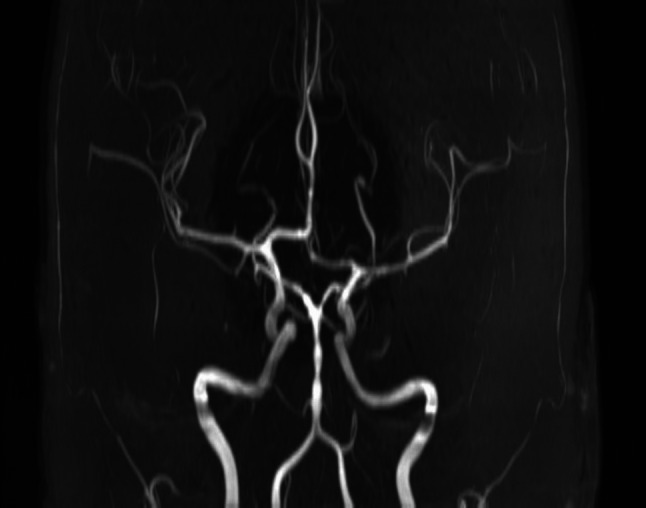
Reconstructed 3D image of an intracranial magnetic resonance (MR) angiography of a patient with tuberculous basilar meningitis. There are multifocal narrowings and irregularities of the basilar artery consistent with consequent basilar artery vasculitis

Time-of-flight MRA (TOF-MRA) is ideal for a rapid non-contrast vascular evaluation or screening, while contrast-enhanced MRA has been found to be superior to TOF-MRA in identifying vessel occlusions and evaluating collateral circulation [37]. Dynamic MRA (dMRA) is another technique that can evaluate collateral circulation better than TOF-MRA [77]; it was also found to detect incomplete occlusions and forward-flow through a thrombus better than TOF-MRA. MRA was also found to be helpful and valuable in evaluating certain vascular etiologies of AIS (Fig. 6). Specifically, vessel-wall imaging MRA (VW-MRA) enables detailed identifications of plaque characteristics to assess its vulnerability [78]. It is a high spatial and contrast resolution MRA with turbo/fast spin-echo sequences which keep signal from blood low and depict thin arterial walls discrete from their surrounding tissues. It can be a problem-solving tool for determining the etiology of stenosis (i.e., atherosclerosis vs. vasculitis vs. dissection). Some studies found VW-MRA useful to investigate the etiology of embolic stroke of undetermined source and cryptogenic stroke [78]. Indeed, Schaafsma et al. showed that VW-MRA determined the etiology of AIS in 55% of patients [79]. Additionally, VW-MRA showing concentric wall thickening and contrast enhancement can be of utility in suspected cases of primary CNS vasculitis with a sensitivity and specificity of 95.2% and 68.8–75% [80].

#### Limitations of MRA

There are practical limitations relating to the use of MRA in AIS, especially TOF-MRA. Overestimation of the degree of stenosis on MRA can lead to inappropriate decision making in the management of AIS [81]. Moreover, it can be difficult to differentiate near-total from total occlusion using MRA alone [82].

### Digital subtraction angiography

**Fig. 7:**
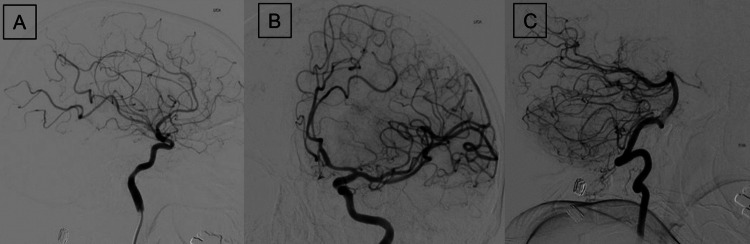
Digital subtraction angiography (DSA) images depicting a case of reversible cerebral vasoconstriction syndrome (RCVS) with multifocal segmental narrowings and dilatations of the intracranial arteries, a “string of beads” appearance

Digital subtraction angiography is a technique that enhances the visualization of blood vessels by subtracting pre-contrast images from post-contrast images. Given its high spatial and temporal resolution, DSA remains the gold standard in the diagnosis of various vasculopathies (Fig. 7). The therapeutic role of DSA is indispensable in AIS. In the setting of AIS, DSA can identify the exact location of an occlusion, guiding endovascular intervention, which is of critical importance in the management of AIS. Indeed, the positive predictive value and negative predictive value of DSA for detecting an LVO reach 91.1% and 95.1%, respectively [83]. Post-endovascular intervention DSA is used to evaluate the degree of revascularization, otherwise known as Thrombolysis in Cerebral Infarction (TICI) score. One study found that DSA imaging of the non-target vessels following endovascular intervention identified previously unknown pathology in 4.4% and led to a change in management in 3.3% of cases [84]. Detailed evaluation of the collateral circulation is another benefit of DSA use in AIS. One study found that DSA-based collateral circulation evaluation in basilar artery stenosis can be helpful in predicting long-term outcomes [85]. However, DSA use is limited by its invasiveness, need for specific expertise, contrast, radiation exposure and availability.

### Transcranial doppler ultrasonography

#### Role as a diagnostic and monitoring tool

Transcranial doppler ultrasonography (TCD) is an inexpensive, non-invasive, real-time hemodynamic monitoring technique that has many clinical and research applications. TCD uses an ultrasound probe and the principle of doppler effect. Ultrasound waves emitted from the probe are transmitted through the bones of the skull and are reflected by moving blood cells within the intracranial vessels back to the probe. The difference in the frequencies between the emitted and reflected waves is known as the doppler shift frequency and is proportional to blood flow velocity. When there is narrowing of a blood vessel, the velocity of blood flow increases to allow for the same volume of blood to flow, and this increase in velocity can be detected by TCD. Furthermore, a TCD signal is a mixture of different doppler frequency shifts as blood flows at different velocities within the cross section of a blood vessel due to laminar flow. This mixture of frequencies can be represented as a spectral display and a spectral analysis representing various parameters including mean flow velocity, peak systolic velocity, end diastolic velocity, and pulsatility index, which can be calculated by the TCD machine. These parameters can be monitored in real-time which makes this modality invaluable especially for continuous monitoring [86]. A recent systematic review found that TCD can be highly accurate in detecting LVO in the acute setting, reaching 85.9% and 99.2% in sensitivity and specificity [87]. This utility can make TCD a useful tool in AIS when other modalities are unavailable. Furthermore, TCD is a useful and cost-effective tool in monitoring patients after endovascular intervention to detect re-occlusion, embolization, or hyper-perfusion [88]. As is the case with other ultrasound-based modalities, TCD can be operator-dependent and requires certain expertise to increase its yield.

#### Role as a potential therapeutic tool

Sonothrombolysis is a term used to describe a process that involves using the mechanical pressure waves of ultrasound to expose the clot’s surface to the circulating thrombolytics, theoretically enhancing their effect [89]. Multiple randomized controlled trials and meta-analyses examined the utility of adding sonothrombolysis to increase the probability of recanalization [90, 91]. These studies showed that sonothrombolysis carried a twofold increase in the odds of complete recanalization compared to thrombolysis alone; clinical outcomes were also improved in patients receiving sonothrombolysis for middle cerebral artery occlusion. Interestingly, the beneficial effect of sonothrombolysis was more pronounced in patients younger than 67 years [90]. TCD is the most common modality used for sonothrombolysis, a promising adjunct to AIS intervention. Future large-scale trials are needed to establish its role and impact as a therapeutic tool.

### Real-life practice considerations

#### Decision-making and clinical context

Technical variables to consider in daily practice include image acquisition protocols and parameters and optimizing image quality while minimizing scanning time is critical in acute stroke evaluation. Table 1 summarizes the characteristics of each modality used in AIS. Variations in acquisition protocols between different institutions can affect the diagnostic accuracy and speed [91]. Standardization of imaging protocols, including slice thickness, contrast timing, and coverage, is important to achieve consistent and reliable results. Efficient neuroimaging workflows are essential for delivering timely care in stroke patients [92]. Integrating imaging with rapid decision-making platforms, such as tele-stroke systems and automated triage protocols, can improve outcomes by accelerating diagnosis and treatment initiation. For example, automated image processing tools like RAPID and Viz.AI can be used to quickly identify ischemic tissue and large vessel occlusions (LVOs), streamlining patient selection for thrombectomy [93]. Understanding the technical limitations of neuroimaging is crucial to avoid misinterpretations. For example, in CT perfusion, poor quality images or motion artifacts may lead to inaccurate measurements of ischemic penumbra. Additionally, pseudo-normalization effects in acute ischemic lesions may occur in later stages of infarction, which could result in misleading conclusions about tissue viability. While neuroimaging is invaluable, it must be interpreted in the context of the patient's clinical presentation [94]. Clinical symptoms, NIH Stroke Scale (NIHSS) score and onset-to-imaging time are key in guiding imaging interpretation and treatment decisions. Over-reliance on imaging alone, without considering clinical context, may lead to suboptimal care.

**Table 1:** Comparison of the different imaging modalities used in acute ischemic stroke evaluation and management

Imaging Modality	Key Strengths	Limitations	Availability	Primary Use in AIS Workflow
Non-contrast CT (NCCT)	Fast, widely available, rules out hemorrhage	Low sensitivity early	Excellent	First-line screening
CT Angiography (CTA)	Detects large vessel occlusion	Requires contrast	Excellent	Vessel status
CT Perfusion (CTP)	Estimates core vs. penumbra	Complex post-processing	Variable	Patient selection for thrombectomy
MRI	Highest sensitivity for ischemia	Limited availability, slower	Moderate	Confirms the diagnosis
MRA	Non-contrast vessel imaging	Longer scan time, overestimation of stenosis degree	Moderate	Vessel status when CTA is unavailable
MRI Perfusion (MRP)	Higher resolution than CTP, especially for posterior fossa structures	Limited availability	Limited	Selection for late-window reperfusion, unknown onset, or equivocal CT findings
DSA	Gold standard for detailed vessel evaluation and guides intervention	Invasive	Limited	Used during intervention
TCD	Real-time vascular physiology monitoring	Operator-dependent	Moderate	Post-intervention monitoring, emerging role in sonothrombolysis
Dual Energy CT	Differentiates contrast-staining from hemorrhage	Limited availability	Moderate	Post-endovascular intervention evaluation
Virtual NCCT	Acquires NCCT and vascular imaging in a single image	Limited availability	Moderate	Potential role in streamlining the AIS workflow with a single image

AIS: acute ischemic stroke; CT: computed tomography; MRI: magnetic resonance imaging; MRA: magnetic resonance angiography; DSA: digital subtraction angiography; TCD: transcranial doppler

#### Direct-to-angio suite paradigm

To enhance the workflow and expedite EVT in patients with LVO, the direct-to-angio suite (DTAS) method has been proposed and studied. The DTAS approach involves bypassing the conventional pathway to imaging and directly transferring the patient to the angiography suite for intervention. Several meta-analyses showed that DTAS resulted in significant reductions in door-to-reperfusion time, by around 35 min compared to the conventional method [6–8] [95–97]. Current evidence suggests that DTAS is a safe, cost-effective and feasible pathway for the management of LVO in AIS. However, DTAS may lead to unnecessary arterial punctures in patients without LVO, and there is significant heterogeneity in the current protocol. Ongoing multicenter trials may shed light on the role of DTAS in LVO and establish its place in guidelines.

### Neuroimaging in adult vs pediatric stroke

In adults, ischemic stroke is most commonly caused by atherosclerosis, embolism, or large vessel occlusion. In contrast, pediatric stroke is often due to conditions such as vasculitis, congenital heart disease, or sickle cell disease, each presenting unique neuroimaging challenges [98]. These etiologies may affect how stroke is visualized on imaging, with conditions like vasculitis showing characteristic vessel wall thickening, which can be evaluated with vessel wall imaging (VWI). Pediatric stroke presents several imaging challenges, including the difficulty of performing high-quality imaging in uncooperative children, smaller vessel sizes that may not be well visualized on standard angiographic techniques, and the need for sedation in many cases. Additionally, the etiology of pediatric stroke can be more diverse, requiring a tailored approach to neuroimaging to address the specific underlying pathology [99]. While MRI (especially diffusion-weighted imaging (DWI)) is the gold standard for pediatric stroke due to its high sensitivity for detecting ischemic changes, the use of CT imaging is generally limited in children due to the risk of radiation exposure [100]. However, CT Angiography can still play a role in evaluating large vessel occlusions in urgent cases.

### Future advancements

Imaging in AIS is advancing swiftly. CT-based modalities are constantly improving to provide MRI-level details in the evaluation of AIS. Advanced perfusion imaging techniques are changing the approach to AIS from a time-based approach to a tissue viability-based one. Another imaging modality that is well-established in coronary interventions but still has not made its way to cerebrovascular disease, is intravascular optical coherence tomography (OCT). Intravascular OCT can provide detailed high-resolutions images of plaques, thrombi, and vascular injury [101]. Finally, artificial intelligence and deep learning techniques are increasingly being utilized to improve the detection time of infarcts, hemorrhage and occlusions [102, 103].

## Conclusions

Imaging modalities in AIS are crucial in the evaluation and management of AIS. However, stroke remains a clinical diagnosis that requires careful clinical-radiological correlations. Each imaging modality in AIS has its own strengths and limitations. Taking these factors into consideration and constantly improving the tools we have at our disposal may ultimately improve patients’ outcomes. It is the combination of these modalities, tailored to the individual patient and hospital capabilities that drives modern stroke care.
